# The *GNB3* c.825C**>**T (rs5443) polymorphism and protection against fatal outcome of corona virus disease 2019 (COVID-19)

**DOI:** 10.3389/fgene.2022.960731

**Published:** 2022-08-09

**Authors:** Birte Möhlendick, Kristina Schönfelder, Christoph Zacher, Carina Elsner, Hana Rohn, Margarethe J. Konik, Laura Thümmler, Vera Rebmann, Monika Lindemann, Karl-Heinz Jöckel, Winfried Siffert

**Affiliations:** ^1^ Institute of Pharmacogenetics, University Hospital Essen, University of Duisburg-Essen, Essen, Germany; ^2^ Department of Nephrology, University Hospital Essen, University of Duisburg-Essen, Essen, Germany; ^3^ Institute for Virology, University Hospital Essen, University of Duisburg-Essen, Essen, Germany; ^4^ Department of Infectious Diseases, University Hospital Essen, University of Duisburg-Essen, Essen, Germany; ^5^ Institute for Transfusion Medicine, University Hospital Essen, University of Duisburg-Essen, Essen, Germany; ^6^ Institute of Medical Informatics, Biometry and Epidemiology, University of Duisburg-Essen, Essen, Germany

**Keywords:** GNB3, rs5443, genetic association, T cell response, G protein, COVID-19, SARS-CoV-2, disease severity

## Abstract

**Background and aims:** Albeit several factors which influence the outcome of corona virus disease (COVID-19) are already known, genetic markers which may predict the outcome of the disease in hospitalized patients are still very sparse. Thus, in this study, we aimed to analyze whether the single-nucleotide polymorphism (SNP) rs5443 in the gene *GNB3*, which was associated with higher T cell responses in previous studies, might be a suitable biomarker to predict T cell responses and the outcome of COVID-19 in a comprehensive German cohort.

**Methods:** We analyzed the influence of demographics, pre-existing disorders, laboratory parameters at the time of hospitalization, and *GNB3* rs5443 genotype in a comprehensive cohort (N = 1570) on the outcome of COVID-19. In a sub cohort, we analyzed SARS-CoV-2-specific T cell responses and associated *GNB3* rs5443 genotypes. We investigated the influence of all factors on COVID-19 fatality in multivariable analysis.

**Results:** We found a younger patient age, normotension or absence of diabetes mellitus or cardiovascular diseases, normal blood cell counts, and low inflammatory markers at hospital admission were protective factors against fatal course of disease. In addition, the rs5443 TT genotype was significantly associated with protection against COVID-19 fatality (OR: 0.60, 95% CI: 0.40–0.92, *p* = 0.02). We also observed significantly increased SARS-CoV-2-specific T cell responses in rs5443 TT genotype carriers (*p* = 0.01). Although we observed a significant association of the factors described previously in univariate analysis, only a younger age of the patients, normal blood cell counts, and the *GNB3* rs5443 TT genotype remained independent predictors against COVID-19 fatality in multivariable analysis.

**Conclusion:** Immutable predictors for COVID-19 fatality are relatively rare. In this study we could show that the TT genotype of the SNP rs5443 in the gene *GNB3* is associated with protection against COVID-19 fatality. It was as well correlated to higher SARS-CoV-2-specific T cell responses, which could result in a milder course of disease in those patients. Based on those observations we hereby provide a further prognostic biomarker, which might be used in routine diagnostics as a predictive factor for COVID-19 mortality already upon hospitalization.

## Introduction

Heterotrimeric guanine-binding proteins (G proteins) transmit signals from the cell surface, trigger intracellular signal cascades, and involve in a wide variety of physiological processes (Klenke et al., 2011). The gene *GNB3* encodes the G protein subunit β3 and is located on chromosome 12p13.31. The ß-subunits are not only the important regulators of the *a*-subunits of G proteins but also intracellular effectors. The synonymous single-nucleotide polymorphism (SNP) rs5443 (c.825C>T; p.S275=) in the gene *GNB3* is associated with several disorders and affects the pharmacodynamics of many different drugs (Klenke et al., 2011). The T allele of this SNP gives rise to the splice variant Gβ3-s, which lacks 123 nucleotides or 41 amino acids. Aberrant splicing results in a dominant gain of function and G protein activation (Siffert et al., 1998). We could show in previous studies that the rs5443 T allele is associated with increased chemotaxis, migration, and proliferation of B lymphoblasts, neutrophils, and T lymphocytes (Virchow et al., 1998; Virchow et al., 1999; Lindemann et al., 2001; Tummala, 2013). Lindemann et al. (2001) could show that CD4^+^ T cell counts are increased in individuals carrying the rs5443 T allele. Therefore, it appears that individuals carrying the T allele show an increased function of their cellular immune system.

Adaptive immune responses, especially those of the T cells, are of major importance in SARS-CoV-2 infection. Virus-specific CD4^+^ and CD8^+^ T cells produce effector cytokines and exert cytotoxic activity in most patients with SARS-CoV-2 infection, whereas neutralizing antibodies directly interfere with viral entry of host cells (Jung and Shin, 2021). Nevertheless, patients with corona virus disease 2019 (COVID-19) not only show lower proportions of SARS-CoV-2-specific CD4^+^ or CD8^+^ T cells but also B cells and NK cells, with increasing disease severity (Huang et al., 2020; Peng et al., 2020; Zeng et al., 2020; Olea et al., 2021). Zeng et al. (2020) observed CD4^+^ T cell lymphopenia in all severe and fatal cases with SARS-CoV-2 infection in their study. Furthermore, the authors could show that prolonged activation and exhaustion of CD8^+^ T cells were associated with COVID-19 severity. In single-cell transcriptomic analyses, encompassing over 80,000 virus-reactive CD8^+^ T single cells, Kusnadi et al. (2021) could show that SARS-CoV-2-reactive CD8^+^ cells exhibited exhausted phenotypes with a decreased capacity to produce cytokines in severely ill COVID-19 patients.

In light of these observations, we hypothesized that the SNP rs5443 in the gene *GNB3* might influence the T cell response in COVID-19 patients as well and, thereby, the outcome of the disease. To answer this question we analyzed the SNP rs5443 in the gene *GNB3* in a comprehensive retrospective German cohort with SARS-CoV-2 infection and its influence upon T cell response and course of COVID-19.

## Methods

### Study participants, recruitment, and outcome of the patients

The study was conducted following the approval of the Ethics Committee of the Medical Faculty of the University of Duisburg-Essen (20-9230-BO) and in cooperation with the West German Biobank (WBE; 20-WBE-088). Written informed consent was obtained from the study patients.

Enrollment started on 11 March 2020, and ended on 18 May 2021. Altogether, 1,570 SARS-CoV-2-positive patients with at least one positive real-time reverse transcription polymerase chain reaction (RT-PCR) test result were consecutively recruited for the study. Follow-up was completed on 30 June 2021, and at that time all patients either were discharged from the hospital as “cured” or had a fatal outcome of the disease. The clinical outcome was defined as follows according to the criteria of the ECDC (European Center of Disease Prevention and Control, 2021)—“mild’: outpatients (*N* = 205); “hospitalized”: inpatients (*N* = 760); “severe”: hospitalized patients admitted to an intensive care unit and/or became dependent on mechanical ventilation (*N* = 292); “fatal” all cases of COVID-19-related deaths during the hospital stay or within a follow-up of 30 days (*N* = 313). In contrast to the ECDC classification, where patients counted up to three times, every patient counted only once, according to the worst clinical outcome observed during the hospital stay in our study. The patients included in this study were of Caucasian origin.

For further statistical analyses, demographic data, medical history, and hematological parameters (erythrocyte, platelet, neutrophil, and lymphocyte counts) at the time of hospital admission were documented for each patient. The medical history included pre-existing disorders of the cardiovascular system (e.g., myocardial infarction, coronary heart disease but not arterial hypertension), arterial hypertension, and diabetes mellitus.

Neutrophil–lymphocyte ratio, platelet–lymphocyte ratio, and systemic immune-inflammation index were calculated as inflammatory markers. The neutrophil-lymphocyte ratio (NLR) is calculated by dividing the number of neutrophils per nanoliter (nl) by the number of lymphocytes per nl from a peripheral blood sample. Similarly, the platelet-lymphocyte ratio (PLR) is calculated, where the number of platelets per nl is divided by the number of lymphocytes per nl in a peripheral blood sample. For the systemic immune-inflammation index (SII), the platelet counts per nl were multiplied by the number of neutrophils per nl and then divided by lymphocyte counts per nl in a peripheral blood sample.

### Interferon-γ ELISpot assay

SARS-CoV-2-specific T cell responses were analyzed in 182 randomly selected SARS-CoV-2-positive patients using interferon-γ (IFN-γ) ELISpot assays as previously described (Schwarzkopf et al., 2021). Briefly, ELISpot stripes containing polyvinylidene difluoride (PVDF) membranes (MilliporeSigma™ MultiScreen™ HTS, Fisher Scientific, Schwerte, Germany) were activated with 50 µl of 35% ethanol for 10 s and washed with distilled water. Plates were then coated for 3 hours with 60 µl of monoclonal antibodies against IFN-γ (10 μg/ml of clone 1-D1K, Mabtech, Nacka, Sweden). Thereafter, ELISpot plates were washed and then blocked with 150 µl AIM-V^®^ (Thermo Scientific, Dreireich, Germany). After 30 min at 37°C, AIM-V^®^ was discarded, and duplicates of 250,000 peripheral blood mononuclear cells (PBMC) were grown in the presence or absence of either PepTivator^®^ SARS-CoV-2 protein S1/S2 (600 pmol/ml, Miltenyi Biotec, Bergisch Gladbach, Germany) in 150 µl of AIM-V^®^. The peptide mix of the S1/S2 protein consists mainly of 15-mer sequences with 11 amino acids overlap, covering the immunodominant sequence domains of the surface glycoprotein of SARS-CoV-2. After 19 h of incubation at 37°C, the ELISpot plates were washed, and captured IFN-γ was detected by incubation for 1 hour with 50 µl of the alkaline phosphatase-conjugated monoclonal antibody against IFN-γ (clone 7-B6-1, Mabtech, Stockholm, Sweden), diluted 1:200 with PBS plus 0.5% bovine serum albumin (BSA). After further washing, 50 µl of nitro blue tetrazolium/5-bromo-4-chloro-3-indolyl-phosphate (NBT/BCIP) was added, and purple spots appeared within 7 min. Spot numbers were analyzed by an ELISpot reader (AID Fluorospot, Autoimmun Diagnostika GmbH, Strassberg, Germany). Mean values of duplicate cell cultures were considered. We determined SARS-CoV-2-specific spots by spot increment, defined as stimulated minus non-stimulated values. Stimulated spot numbers > 3-fold higher than negative (unstimulated) controls combined with an increment value of >3 to the antigen were considered positive. Of note, the negative controls reached a mean value of less than one spot.

### Genotyping of *GNB3* rs5443 (c.825C>T)

Genomic DNA was extracted from 200 µl EDTA-blood using the QIAamp^®^ DNA Blood Mini Kit (Qiagen, Hilden, Germany). Polymerase chain reaction (PCR) was performed with 2 µl genomic DNA and 30 µl *Taq* DNA-Polymerase 2x Master Mix Red (Ampliqon, Odense, Denmark), with the following conditions: initial denaturation 94°C for 3 min; 35 cycles with denaturation 94°C for 30 s, annealing at 66°C for 30 s, and elongation 72°C for 30 s each; final elongation 72°C for 10 min (forward primer: 5′ GCT GCC CAG GTC TGA TCC C 3' and reverse primer 3′ TGG GGA GGG TCC TTC CAG C 5′). PCR products were digested with *Bse*DI (Thermo Scientific, Dreireich, Germany), and restriction fragments were analyzed by agarose gel electrophoresis. The various genotype results from restriction fragment length polymorphism (RFLP)-PCR were validated by Sanger sequencing.

### Statistical analyses

Correlation of demographics (sex and medical history) and outcome of COVID-19 were calculated using Pearson’s chi square (χ^2^) statistics using the Baptista–Pike method for the odds ratio (OR) and 95% confidence interval (CI). One-way analysis of variance (ANOVA) was performed using the Kruskal–Wallis test with Dunn’s multiple comparison to assess the influence of age, hematological parameters, or inflammatory markers on COVID-19 severity. To calculate thresholds for the laboratory values, which correlate with fatal course of disease receiver operating characteristic (ROC) analysis, Youden`s J statistic was performed.

The number of patients with fatal outcome of disease, for whom IFN-γ ELISpot analyses could be performed, was relatively small. Thus, we defined additional groups to perform statistical analyses to estimate the influence of the T cell response on COVID-19 severity in our cohort. Therefore, patients from the categories “mild” and “hospitalized” were grouped together to the group “moderate,” whereas the patients with “severe” and “fatal” COVID-19 were consolidated to the group “serious.” The differences in T cell responses as analyzed by IFN-γ ELISpot between patients with “moderate” and “serious” COVID-19 was estimated by Mann–Whitney test.

Hardy–Weinberg equilibrium (HWE) was calculated using Pearson’s chi square (χ^2^) goodness of fit test, and samples were considered as deviant from HWE at a significance level of *p* < 0.05.

For genetic association, we calculated OR and 95% CI by Pearson’s chi square (χ^2^) statistics using the Baptista–Pike method for OR and 95% CI, respectively. *p*-values are reported two-sided, and values of <0.05 were considered significant. One-way analysis of variance (ANOVA) was performed using Kruskal–Wallis test with Dunn’s multiple comparison test to determine the influence of *GNB3* rs5443 genotype on T cell response as measured by IFN-γ ELISpot assay.

Multivariable analysis was performed to estimate independency of the variables age, sex, medical history, laboratory parameters, and *GNB3* rs5443 genotypes by stepwise Cox regression (likelihood ratio test, backward).

## Results

From 11 March 2020 to 30 June 2021, we enrolled and studied 1,570 SARS-CoV-2-positive patients to determine the association of the SNP rs5443 in the gene *GNB3*, with severity of COVID-19. In a sub group of patients (*N* = 182), who were representative for all severity groups, we additionally analyzed the T cell response to SARS-CoV-2-specific antigens. The demographics and clinical characteristics of the patients are summarized in Table 1. We observed that about 20% of all patients (inpatients and outpatients) and 23% of the hospitalized patients had a fatal outcome of COVID-19. With increasing severity of the disease, we found significantly more elderly and male patients and those who had arterial hypertension, cardiovascular disorders, or diabetes mellitus as pre-existing medical disorders (Table 1). The number of platelets, erythroctytes, and lymphocytes decreased significantly, whereas the neutrophil counts increased with disease severity (*p* < 0.0001, ANOVA). Regarding the inflammatory markers, NLR, PLR, and SII, we observed significantly higher values with increasing severity of COVID-19 as well.

**TABLE 1:** Demographics, clinical characteristics, and outcome of the disease in SARS-CoV-2-positive patients. Classification according to the COVID-19 surveillance report of the ECDC: category “mild” is a case that has not been reported as hospitalized or dead. A “severe” case has been admitted to intensive care and/or required mechanical respiratory support. All values are given in medians and interquartile ranges (IQR), except from sex and medical history, which are reported in absolute counts and percentages.

Characteristics	All patients (*N* = 1570)	Mild(*N* = 205)	Hospitalized(*N* = 760)	Severe(*N* = 292)	Fatal(*N* = 313)	*p*-value
Age–years	62.0 (49.0–76.0)	47.0 (34.5–64.0)	62.0 (48.3–76.0)	59.0 (50.0–70.0)	71.0 (59.5–82.0)	*p* < 0.0001
Male sex	910 (58.0)	107 (52.2)	416 (54.7)	185 (63.4)	202 (64.5)	*p* = 0.002
Medical history						
Cardiovascular system^a^	547 (34.8)	11 (5.4)	257 (33.8)	111 (38.0)	168 (53.7)	*p* < 0.0001
Arterial hypertension	748 (47.6)	29 (14.1)	373 (49.1)	149 (51.0)	197 (62.9)	*p* < 0.0001
Diabetes mellitus	404 (25.7)	14 (6.8)	214 (28.2)	76 (26.0)	100 (31.9)	*p* = 0.001
Hematological parameters						
Erythrocytes/nl	4.4 (3.8–4.8)	4.6 (4.2–4.9)	4.4 (4.0–4.9)	4.4 (3.8–4.8)	4.0 (3.4–4.6)	*p* < 0.0001
Platelets/nl	202.0 (156.0–260.0)	204.0 (164.0–270.5)	205.0 (157.0–255.0)	209.0 (169.0–292.0)	189.0 (135.0–242.0)	*p* < 0.0001
Neutrophils/nl	4.9 (3.1–7.5)	3.7 (2.7–5.1)	3.9 (2.6–5.8)	6.3 (4.2–9.3)	7.7 (5.2–11.7)	*p* < 0.0001
Lymphocytes/nl	0.9 (0.7–1.3)	1.1 (0.9–1.5)	1.0 (0.7–1.4)	0.8 (0.6–1.1)	0.7 (0.5–1.1)	*p* < 0.0001
Inflammatory markers						
NLR	5.0 (2.9–9.9)	3.1 (2.1–4.7)	3.8 (2.4–6.2)	7.9 (4.5–13.0)	11.1 (6.0–18.5)	*p* < 0.0001
PLR	217.8 (151.1–326.7)	176.3 (139.1–268.9)	197.7 (140.7–285.5)	269.8 (181.4–418.4)	252.6 (162.9–414.2)	*p* < 0.0001
SII	1031.0 (523.9–2206.0)	717.2 (385.7–1055.0)	769.6 (399.8–1417.0)	1680 (921.9–3466.0)	1917.0 (1010.0–4019.0)	*p* < 0.0001

a Cardiovascular system: for example, myocardial infarction, coronary heart disease but not arterial hypertension. Abbreviations: nl = nanoliter; NLR, neutrophil–lymphocyte ratio; PLR, platelet–lymphocyte ratio; SII, systemic immune-inflammation index.

### SARS-CoV-2-specific T cell response and *GNB3* rs5443 genotype

**FIGURE 1:**
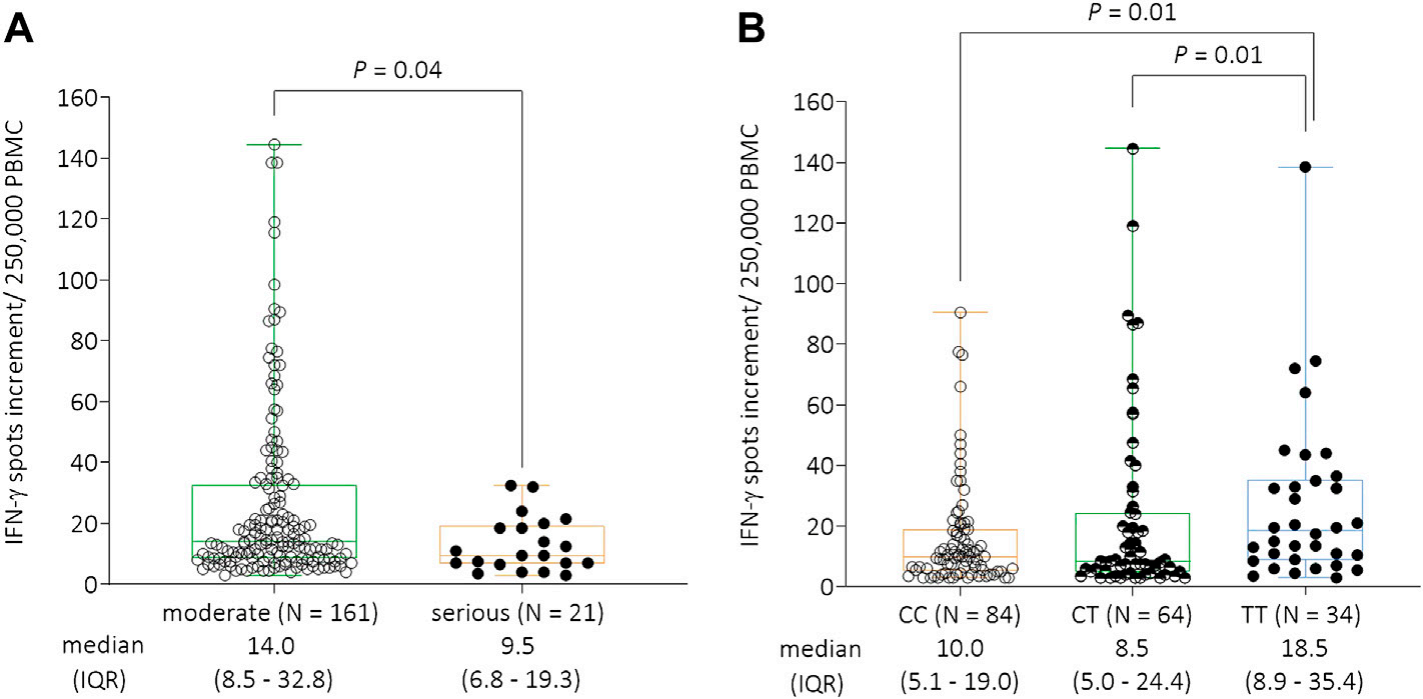
**(A)** IFN-γ ELISpot responses to S1/S2 protein of SARS-CoV-2 per 250,000 peripheral blood mononuclear cells stratified by COVID-19 severity. Due to the low number of cases in the individual groups, patients from the categories “mild” and “hospitalized” were grouped together to the group “moderate,” whereas the patients with “severe” and “fatal” COVID-19 were consolidated to the group “serious.” There was a significant decline of IFN-γ spots increment comparing the “serious” (*N* = 21, median = 9.5, and IQR = 6.8–19.3) to the “moderate” (*N* = 161, median = 13.5, and IQR = 8.0–35.0) groups (*p* = 0.04). **(B)** IFN-γ ELISpot responses to S1/S2 protein of SARS-CoV-2 per 250,000 peripheral blood mononuclear cells stratified by the *GNB3* rs5443 genotype. Individuals with the TT genotype had significantly higher spots increment (median = 18.5, and IQR = 8.9–35.4) compared to CT (median = 8.5, IQR = 5.0–24.4, and *p* = 0.01) or CC genotype carriers (median = 10.0, IQR = 5.1–19.0, and *p* = 0.01). Abbreviations: IFN-γ = interferon gamma; PBMC = peripheral blood mononuclear cells; IQR = interquartile range.

In 182 patients, we performed IFN-γ ELISpot assays to determine T cell response to SARS-CoV-2-specific antigens. We were able to analyze patients from all severity groups: “mild” (*N* = 79); “hospitalized” (*N* = 82); “severe” (*N* = 17), and “fatal” (*N* = 4). The number of patients with fatal outcome of disease, for whom IFN-γ ELISpot analyses could be performed, was relatively small. Thus, we defined additional groups to perform statistical analyses to estimate the influence of the T cell response on COVID-19 severity in our cohort. Therefore, patients from the categories “mild” and “hospitalized” were grouped together to the group “moderate,” whereas the patients with “severe” and “fatal” COVID-19 were consolidated to the group “serious.” We observed a significant decline of spots increment in the IFN-γ ELISpot assay comparing the “serious” group (*N* = 21, median = 9.5, and IQR = 6.8–19.3) to the “moderate” group (*N* = 161, median = 14.0, IQR = 8.5–32.8, *p* = 0.04, Figure 1A).

In a next step, we analyzed the influence of *GNB3* rs5443 genotypes on IFN-γ production against SARS-CoV-2-specific antigens. Here, we found a significant increase of IFN-γ spots increment in TT genotype carriers (median = 18.5 and IQR = 8.9–35.4) compared to those with CC genotype (median = 10.0, and IQR = 5.1–19.0) or CT genotype (median = 8.5, and IQR = 5.0–24.4) (both *p* = 0.01, respectively, Figure 1B).

### *GNB3* rs5443 as a protective factor against COVID-19 fatality

Overall, the observed genotypes for *GNB3* rs5443 were compatible with HWE in patients with “mild” (*p* = 0.66), “hospitalized” (*p* = 0.07), “severe” (*p* = 0.52), and “fatal” (*p* = 0.28) SARS-CoV-2 infection. Genotype distributions for all patients and the different groups according to severity of SARS-CoV-2 infection are shown in Table 2. Notably, we observed very similar rs5443 T allele frequencies (35%–36%) in all groups, except from those patients with a “fatal” outcome of COVID-19 (29%). Thus, we estimated, whether T allele or TT genotype carriers might be protected more effectively against fatal outcome of the disease. We found a significant association for protection against COVID-19 fatality in rs5443 TT genotype carriers comparing all patients (“mild,” “hospitalized,” and “severe”) with SARS-CoV-2 infection and with those who died from COVID-19 (OR: 0.60, 95% CI: 0.40–0.92; *p* = 0.02, Table 3).

**TABLE 2:** *GNB3* rs5443 (c.825C>T) genotype distribution among all patients with SARS-CoV-2 infection and subdivided according to the severity of COVID-19.

	All patients (*N* = 1,570)	Mild(*N* = 205)	Hospitalized(*N* = 760)	Severe(*N* = 292)	Fatal(*N* = 313)
*GNB3* rs5443 CC	700 (44.6)	89 (43.4)	330 (43.4)	121 (41.4)	160 (51.1)
*GNB3* rs5443 CT	666 (42.4)	90 (43.9)	324 (42.6)	130 (44.5)	122 (39.0)
*GNB3* rs5443 TT	204 (13.0)	26 (12.7)	106 (13.9)	41 (14.0)	31 (9.9)
Minor allele frequency (T)	0.34	0.35	0.35	0.36	0.29

**TABLE 3:** Protective factors against COVID-19 fatality. Abbreviations: nl = nanoliter; NLR = neutrophil-lymphocyte ratio; PLR = platelet-lymphocyte ratio; SII = systemic immune-inflammation index; OR = odds ratio; CI = confidence interval, NS = not significant in stepwise multivariable analysis.

	Univariate analysis		Multivariable analysis	
**Factor**	**OR (95% CI)**	***p*** **-value**	**Or ([95% CI)**	***p*** **-value**
Age (<62 years)	0.35 (0.27–0.45)	<0.0001	0.47 [0.34–0.64)	<0.0001
Sex (female)	0.71 (0.55–0.92)	0.01	NS	NS
Absence of				
Diseases of cardiovascular system	0.41 (0.32–0.53)	<0.0001	NS	NS
Arterial hypertension	0.53 (0.41–0.68)	<0.0001	NS	NS
Diabetes mellitus	0.75 (0.57–0.98)	0.04	NS	NS
Hematological parameters				
Erythrocytes (≥4.0/nl)	0.27 (0.21–0.34)	<0.0001	0.70 (0.52–0.94)	0.02
Platelets (≥133.5/nl)	0.40 (0.29–0.54)	<0.0001	0.42 (0.30–0.60)	<0.0001
Neutrophils (≤6.6/nl)	0.28 (0.21–0.36)	<0.0001	0.32 (0.23–0.45)	<0.0001
Lymphocytes (≥0.9/nl)	0.41 (0.32–0.53)	<0.0001	0.55 (0.41–0.74)	<0.0001
Inflammatory markers				
NLR (<7.3)	0.24 (0.18–0.31)	<0.0001	NS	NS
PLR (<224.6)	0.60 (0.46–0.78)	<0.0001	NS	NS
SII (<1206.4)	0.32 (0.24–0.42)	<0.0001	NS	NS
*GNB3* rs5443 TT genotype	0.60 (0.40–0.92)	0.02	0.65 (0.44–0.96)	0.03

Thereupon, we performed multivariable analysis to analyze the independence of the *GNB3* rs5443 TT genotype in comparison to the other predictive parameters: age, pre-existing disorders, hematological parameters, and inflammatory markers. We performed ROC analysis and Youden`s statistic for the numeric variables to estimate a threshold above which the risk for COVID-19 fatality significantly decreased. We found that a younger patient age (<62 years; *p* < 0.0001), erythrocyte (≥4.0/nl; *p* = 0.02), platelet (≥133.5/nl; *p* < 0.0001), neutrophil (<6.6/nl; *p* < 0.0001), and lymphocyte (≥0.9/nl; *p* < 0.0001) counts above these respective thresholds at the time of admission to hospital, and the *GNB3* rs5443 TT genotype (*p* = 0.03) remained independent predictors for protection against COVID-19 fatality (Table 3).

## Discussion

Remarkably, we observed that the TT genotype of the SNP rs5443 in the gene *GNB3* was associated with a higher T cell response as estimated by IFN-γ ELISpot assay in our patients. We could not find an association of *GNB3* genotype to lymphocyte or T cell counts. Thus, it seems that the increased T cell response in TT genotype carriers might be related to an increased activation of T cells. Early development of CD8^+^ T cell responses is correlated to a more effective viral clearance and a mild course of COVID-19. Patients with severe disease display early onset of inflammation as well as delayed and relatively excessive adaptive immune response (Moss, 2022). The SNP rs5443 in the gene *GNB3* was not only correlated to higher T cell responses but also to a significantly reduced risk for COVID-19 fatality in our study in univariate and multivariable analyses.

The underlying mechanism of the influence of *GNB3* genotype on T cell response remains elusive. Juno (2014) could show that *GNB3* TT genotype carriers had a significantly lower *LAG-3* gene expression. The *LAG-3* (lymphocyte activation gene 3) gene is localized on chromosome 12 nearby to *GNB3*, nevertheless there are no SNPS in the gene *LAG-3* in high linkage disequilibrium with rs5443, which could be causative for the different gene expression in *GNB3* TT genotype carriers. LAG-3 was found to be expressed on dysfunctional or exhausted T cells in chronic viral infections and correlated with severity of the infection (Blackburn et al., 2009; Richter et al., 2010). Further studies are needed to analyze whether a reduced *LAG-3* expression is responsible for the T cell activation in *GNB3* TT genotype carriers.

We found that the comorbidities arterial hypertension, other disorders of the cardiovascular system and diabetes mellitus were associated with COVID-19 fatality in univariate analysis. This has already been extensively described in a multitude of studies and meta-analyses (Zhou et al., 2020). A variety of other factors, for example, age, sex, or laboratory parameters, have also been identified to influence the course of COVID-19 (Hobohm et al., 2022). The infection-fatality ratio of COVID-19 significantly increases through ages 30, 60, and 90 years (COVID-19 Forecasting Team, 2022). Thus, we observed that a younger age (<62 years) was an independent protective factor against COVID-19 fatality in our study as well. Nevertheless, we could not confirm the independent influence of other consistent factors, like sex or pre-existing disorders, in a multivariable analysis.

Normal cell counts of lymphocytes and platelets upon hospital admission are associated with a significantly reduced risk for fatal outcome of COVID-19. Impaired adaptive immune responses as reflected by low counts of white blood cells together with augmented inflammation serve as a good predictor for the course of the disease (Qin et al., 2022). In our study, we noticed impaired white blood cell counts in individuals with severe COVID-19 as well. Several studies could show that the inflammatory markers NLR, PLR, and SII determined upon hospital admission are good predictive markers for in-hospital mortality (Fois et al., 2020; Wang et al., 2021; Sarkar et al., 2022). We also found a significant association for COVID-19 fatality and high NLR, PLR, and SII in the univariate analysis in our study. Nonetheless, those markers did not reach statistical significance in the multivariable analysis. Therefore, it seems even more important to find persistent markers that can predict the course of COVID-19 disease.

Together with a younger patient age, a normal white blood cell count at hospital admission, the *GNB3* rs5443 TT genotype remained an independent protective factor against COVID-19 fatality in our study. Immutable predictors are still relatively rare, thus analyses of genetic host factors might be useful in predicting severity, which could be implemented in routine diagnostics.

## Data Availability

The original contributions presented in the study are included in the article/supplementary materials; further inquiries can be directed to the corresponding author.
